# First report on dog bite epidemiology and Rabies diagnosis in stray dogs: a one health study from Puducherry

**DOI:** 10.3389/fvets.2025.1642231

**Published:** 2025-08-29

**Authors:** Abhiram Naidu Killada, V. Bhanu Rekha, Shrikrishna Isloor, V. J. Ajay Kumar, P. Vijayalakshmi, Nithya Quintoil, Sundarachelvan Subramanian, Shraddha Singh, Neha M. Banakar, G. Deepan, S. Ravivarman

**Affiliations:** ^1^Department of Veterinary Public Health, Rajiv Gandhi Institute of Veterinary Education and Research (RIVER), Pondicherry University, Puducherry, India; ^2^KVAFSU-CVA Rabies Diagnostic Laboratory, WOAH Reference Laboratory for Rabies, Department of Veterinary Microbiology, Veterinary College, Karnataka Veterinary, Animal and Fisheries Sciences University (KVAFSU), Bengaluru, India; ^3^Department of Veterinary Medicine, Rajiv Gandhi Institute of Veterinary Education and Research (RIVER), Pondicherry University, Puducherry, India; ^4^Bark India Charitable Trust, Villupuram, India

**Keywords:** rabies, DFA, one health, one-step RT-PCR, dRIT

## Abstract

**Introduction:**

Rabies is a fatal zoonotic disease transmitted primarily through dog bites. Monitoring bite incidence and reliable rabies diagnostic methods are crucial for effective rabies control, especially in endemic regions. So, the present study was conducted to estimate the burden of dog bite cases in humans and animals, and to confirm the presence of rabies using various diagnostic tests among dogs in Puducherry, India.

**Materials and methods:**

A regional descriptive study was done with objectives to collect the data on human and animal dog bite cases from 2020 to 2023 and 25 brain samples from dogs suspected of rabies were collected post-mortem using the foramen magnum method. Samples were tested using Lateral Flow Assay (LFA), Direct Fluorescent Antibody test (DFA), Direct Rapid Immuno-histochemistry Test (dRIT), and One-step RT-PCR. Sensitivity and specificity of these tests were compared using DFA as the gold standard to estimate the burden of rabies in stray dogs.

**Results:**

Dog bite cases in humans increased from 16,652 in 2020 to 20,063 in 2023, with December consistently reporting the highest average number of bites (1787.25 cases). Animal bite cases rose from 948 in 2022 to 1,131 in 2023, affecting dogs (56.1%), goats (30.7%), cattle (14.3%), and cats (0.9%). Of the 25 dog brain samples tested, 19 (76%) were rabies positive. LFA, dRIT, and RT-PCR demonstrated 100% sensitivity and specificity compared to DFA. Rabies cases were nearly equal in females (52.6%) and males (47.4%), with 68.4% occurring in dogs under 3 years. The highest monthly cases were observed in May and October, with Reddiarpalayam and Lawspet being the most affected regions.

**Discussion and conclusion:**

The study reveals a concerning rise in dog bite cases and confirms the utility of LFA, dRIT, and RT-PCR as reliable alternatives to DFA for rabies diagnosis. Enhanced surveillance, public awareness, and vaccination programs are essential to control rabies in Puducherry.

## 1 Introduction

Rabies is one of the deadliest zoonotic diseases, with an almost 100% fatality rate once clinical symptoms appear (1). Globally, it is classified into urban rabies, where domestic dogs and cats serve as primary reservoirs, and sylvatic rabies, maintained by wildlife such as foxes, wolves, and bats (2). The disease remains a significant public health concern, causing an estimated 59,000 human deaths annually, with 95% occurring in Asia and Africa, particularly in resource-limited settings (3).

India is highly endemic for rabies, with an estimated 20,000 human deaths annually, accounting for nearly 29% of the global rabies burden (4). However, the Andaman and Nicobar Islands and Lakshadweep have historically remained rabies-free (5). Various epidemiological studies have attempted to estimate rabies mortality in India. A verbal autopsy survey within the Million Death Study (MDS) projected 12,700 rabies deaths in 2005 (6), while a probability-based model estimated 16,450 deaths in 2010 (7). Dog bites remain the primary cause of human rabies cases in India (97%), followed by cats (2%) and wildlife (1%) (8). The annual incidence of animal bites is estimated at 1.7% (17.5 million cases per year), with reported cases rising from 4.2 million in 2012 to 7.4 million in 2018 under the Integrated Disease Surveillance Programme (IDSP).

To combat this escalating burden, the World Health Organization (WHO), the World Organization for Animal Health (WOAH), the Food and Agriculture Organization (FAO), and the Global Alliance for Rabies Control (GARC) have launched the United Against Rabies Collaboration, aiming for zero human deaths from dog-mediated rabies by 2030 (7). Achieving this target requires rapid, accurate, and accessible diagnostic methods to enhance surveillance and optimize post-exposure prophylaxis administration (9).

The Direct Fluorescent Antibody (DFA) test, developed in 1958, remains the gold standard for rabies diagnosis in both humans and animals and is recommended by WHO and WOAH (2). The test is typically performed on brain tissue, particularly from the brainstem and hippocampus, collected from animals. However, alternative diagnostic methods have gained traction due to DFA’s reliance on specialized equipment and technical expertise. The Lateral Flow Assay (LFA) offers a rapid, user-friendly alternative, providing results within 10 min without requiring advanced laboratory infrastructure (10). The Direct Rapid Immunohistochemical Test (dRIT), developed by the Centers for Disease Control and Prevention (CDC), targets rabies virus (RABV) nucleoprotein in brain tissue, delivering results in under an hour while being adaptable to field settings (11). Additionally, molecular techniques such as reverse transcriptase-polymerase chain reaction (RT-PCR) and real-time RT-PCR demonstrate superior sensitivity and specificity, making them valuable tools for rabies surveillance and variant characterization (12).

In this study, we attempted to analyze the reported dog bite cases in both animals and humans and evaluate the diagnostic performance of LFA, DFA, dRIT, and RT-PCR in suspected rabies cases in dogs from the Puducherry region of India. To the best of our knowledge, this is the first study evaluating rabies diagnostics in dogs from the Puducherry region.

## 2 Materials and methods

### 2.1 Study area

The present study was conducted in Puducherry, India, located in the southeastern coastal region of the country at coordinates 11° 94’N and 79° 80′E and at an elevation of 15 m above mean sea level (13).

### 2.2 Collection of dog bite case data

A regional descriptive study was done to collect the data on dog bite cases reported in both humans and animals from the Puducherry region.

The details of the number of dog bite cases in humans from January 2020 to December 2023 in Puducherry have been collected from the Integrated Disease Surveillance Programme (IDSP), Puducherry.

The details of the dog bite cases in animals from January 2022 to December 2023 were collected from the Veterinary Clinical Complex, Rajiv Gandhi Institute of Veterinary Education and Research (RIVER), Kurumbapet, Puducherry and various Veterinary Dispensaries under the Department of Animal Husbandry and Animal Welfare, Government of Puducherry. The details of all the Veterinary Dispensaries are listed in Supplementary Table 1.

### 2.3 Brain sample collection from dogs

A total of 25 dog brain samples were collected over 1 year (January 2023–December 2023) from suspected dead rabid animals from various parts of the Puducherry region. A convenience sample of 25 brain specimens was collected based on availability during the study period.

Brain samples were collected from the Veterinary Clinical Complex, Veterinary dispensaries, municipalities and various non-governmental organizations (NGO’s) using the Foramen Magnum (section 2.4) method of brain sampling (Supplementary Table 2). All the samples were collected aseptically and using personal protective equipment. The samples were triple-packed and transported in ice to the Department of Veterinary Public Health and Epidemiology, RIVER, for storage and further processing of samples, which were done at KVAFSU-CVA Rabies Diagnostic Laboratory, WOAH reference laboratory for rabies, Department of Veterinary Microbiology, Veterinary College, Hebbal, Bengaluru, KVAFSU.

### 2.4 Brain sample collection through the Foramen magnum approach

Initially, the carcass was kept on the post-mortem table in the lateral recumbency with the head flexed ventrally. A deep incision was made just behind the nuchal crest of the occipital bone, severing the skin, cutaneous fascia, cervicoscutularis muscle, splenius, brachiocephalicus muscles and the insertion point of the nuchal ligament to expose the occipito-atlantal joint. The joint was then dislocated using a sharp, disposable scalpel blade. This exposed the Foramen magnum, which seats some parts of the Pons, Medulla oblongata and major parts of the brainstem. An artificial insemination (AI) sheath was cut to the required size and connected to a disposable syringe was inserted deeply into the Foramen magnum, and the tissue from the brain stem was aspirated into the AI sheath (14).

### 2.5 Diagnostic tests

#### 2.5.1 Lateral flow assay (LFA)

The kit used for this study was Anigen, Rapid Rabies Ag Test Kit produced by Bionote, Inc. (Hwaseong-si, Korea). Brain tissue (brainstem and hippocampus) collected through the foramen magnum approach was homogenized and directly diluted at a 1:10 ratio in the assay buffer provided with the diagnostic kit. The test device was removed from the foil pouch and placed on a flat, dry surface. The sample was taken from the specimen tube using a disposable dropper provided in the kit. Four drops of the sample were added into the sample hole using the disposable dropper. The result of the test was observed within 5–10 min. The presence of two bands in the result window at position “T” (Test sample) and “C” (Control) indicated the presence of the virus (15).

#### 2.5.2 Direct fluorescent antibody test (DFA)

An impression smear (~10 mm diameter) was prepared from two brain regions (brainstem and cerebellum) on clean, dry, pre-rinsed glass slides. Control slides were similarly prepared using a normal dog brain (negative control) and rabies-infected brain samples confirmed by DFA and RT-PCR (positive control). The smears were air-dried (10–15 min) at room temperature, fixed in chilled acetone (−80 °C, 1 h), and air-dried again. Each smear was then incubated with 40 μL of FITC-labeled anti-rabies nucleoprotein conjugate (1,100 in 1x PBS, pH 7.4) (Merck Millipore, Temecula, CA, USA) at 37 °C for 60 min in a high-humidity chamber. After staining, excess conjugate was drained, and slides were rinsed and soaked in PBS (3–5 min). Observation was made under an inverted fluorescent microscope (400 nm) (Carl Zeiss AG, Göttingen, Germany) within 2 h, confirming rabies positivity by the presence of granular intra-cytoplasmic apple-green fluorescence of aggregated nucleocapsids (3).

#### 2.5.3 Direct rapid immunohistochemistry test (dRIT)

Impressions of suspected brain tissues, from the brainstem due to high antigen concentration, were made on labeled glass slides. All steps were conducted at room temperature. Smears were air-dried (5 min), fixed in 10% buffered formalin (10 min), and rinsed in Tween 20 and PBS (TPBS) to remove excess fixative. They were then immersed in 3% hydrogen peroxide (10 min) for cleaning, followed by another TPBS rinse. Excess buffer was shaken off, and the area around the smear was blotted dry. Slides were placed in a humid chamber, and biotinylated anti-rabies mAb was applied to the impressions, incubated for 10 min, then rinsed in TPBS. Streptavidin-peroxidase complex was added and incubated (10 min), followed by another TPBS rinse. A freshly prepared amino ethyl carbazole solution was applied (10 min), and slides were rinsed in distilled water. After blotting dry, smears were counterstained with 1:2 diluted hematoxylin (2 min), thoroughly washed, and mounted with a water-soluble medium under a coverslip. Observations under a light microscope at 20x magnification for scanning and 40x for detailed inspection revealed rabies virus antigen as red inclusions against a blue neuronal background (3).

#### 2.5.4 Molecular confirmation of rabies virus

Total RNA was extracted from brain tissues using the HiPurA® Viral RNA Purification Kit (HiMedia) with slight modifications. The RT-PCR was carried out directly from isolated RNA samples using the QIAGEN One-step RT-PCR, and the amplified PCR products were separated and visualized by gel electrophoresis in a 1.5% agarose gel. The primer used in the study is given in Table 1.

**Table 1:** Details of the primer for one-step RT-PCR.

Sl. No	Primer	Sequence 5’→3’	Amplicon size(bp)	Reference
1.	JW 12 F	ATGTAACACCTCTACAATG	605 bp	Prabhu et al. (3)
	JW 6 R	CAATTAGCACACATTTTGTG		

### 2.6 Statistical analysis of different diagnostic tests

To compare the sensitivity and specificity of the Lateral Flow Assay (LFA), Direct Rapid Immunohistochemistry Test (dRIT), and One-step RT-PCR with the Direct Fluorescent Antibody (DFA) test as the standard, the statistical formula described by Thrusfield (16) was used.


Sensitivity(%)=a/(a+c)×100

Where:

𝑎 = Number of samples positive by both standard and comparison tests.

𝑐 = Number of samples positive by the standard test but negative by the comparison test.


Specificity(%)=d/(b+d)×100

Where:

𝑑 = Number of samples negative by both standard and comparison tests.

𝑏 = Number of samples negative by the standard test but positive by the comparison test.

## 3 Results

### 3.1 Dog bite cases in humans

**Figure 1:**
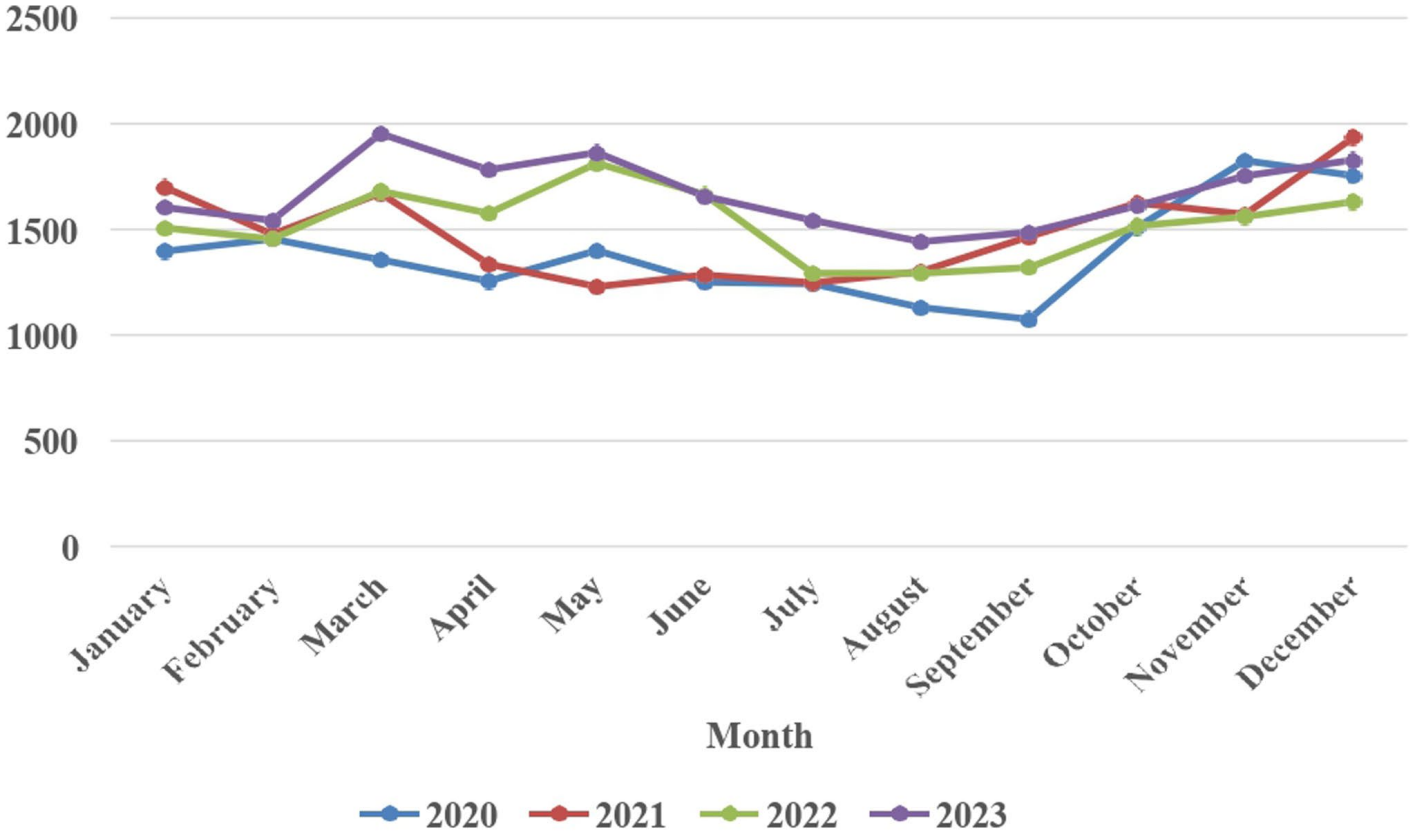
Monthly trends of dog bite cases in humans from 2020 to 2023.

Over the period from 2020 to 2023, there has been a steady rise in the incidence of dog bite cases, with reported numbers increasing from 16,652 in 2020 to 20,063 in 2023 (20.5% increase over 4 years, average annual increase of 5.1%). The average monthly incidence of dog bite cases in humans across the years 2020 to 2023 revealed distinct patterns. Notably, December emerged as the month with the highest average number of cases, reaching 1787.25. In contrast, August exhibited the lowest average, recording 1,291 average cases. The monthly distribution of dog bite cases in humans from 2020 to 2023 is illustrated in Figure 1. Despite the absence of a discernible specific trend, a comparative analysis between the years 2020 and 2023 reveals a substantial surge in the number of bite cases each month.

### 3.2 Dog bite cases in animals

Between January 2022 and December 2023, a total of 2079 cases of dog bites were reported in animals. Notably, there was a slight upturn in the number of cases, increasing from 948 in 2022 to 1,131 in 2023.

**Figure 2:**
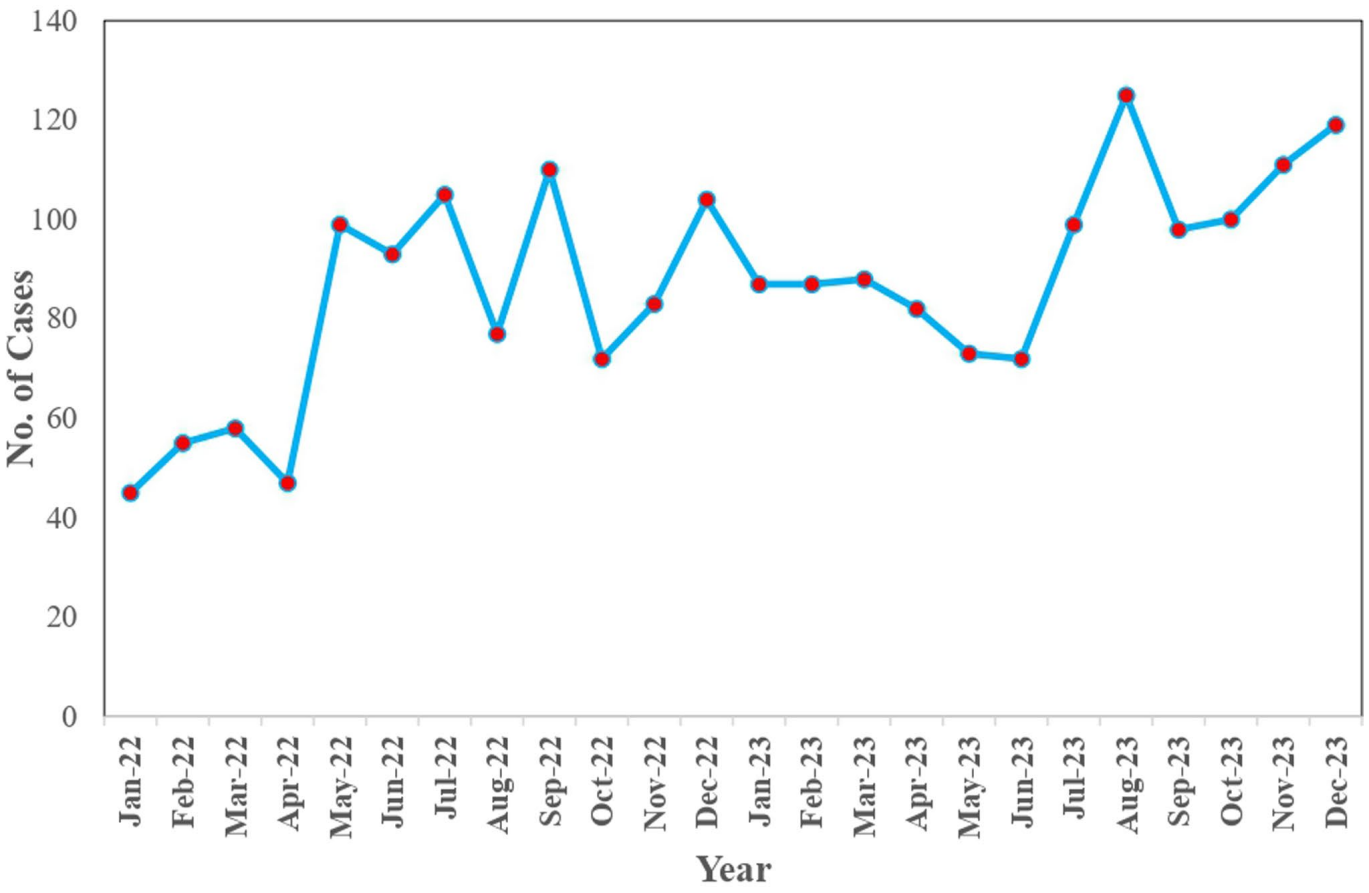
Monthly distribution of dog bite cases in animals (2022–2023).

During this period, the most affected species of animals were dogs with 54.10% (1,123/2079) of the cases, followed by goats, cattle and cats with 30.70% (640/2079), 14.30% (297/2079) and 0.90% (19/2079) cases, respectively. When the month-wise distribution of the dog bite cases was observed, there was a gradual increase in the number of dog bite cases from January 2022 to December 2023, with August 2023 having the highest number of cases, 125, and April 2022 being the lowest with 39 cases (Figure 2).

### 3.3 Diagnostic tests

#### 3.3.1 Lateral flow assay test (LFA)

**Figure 3:**
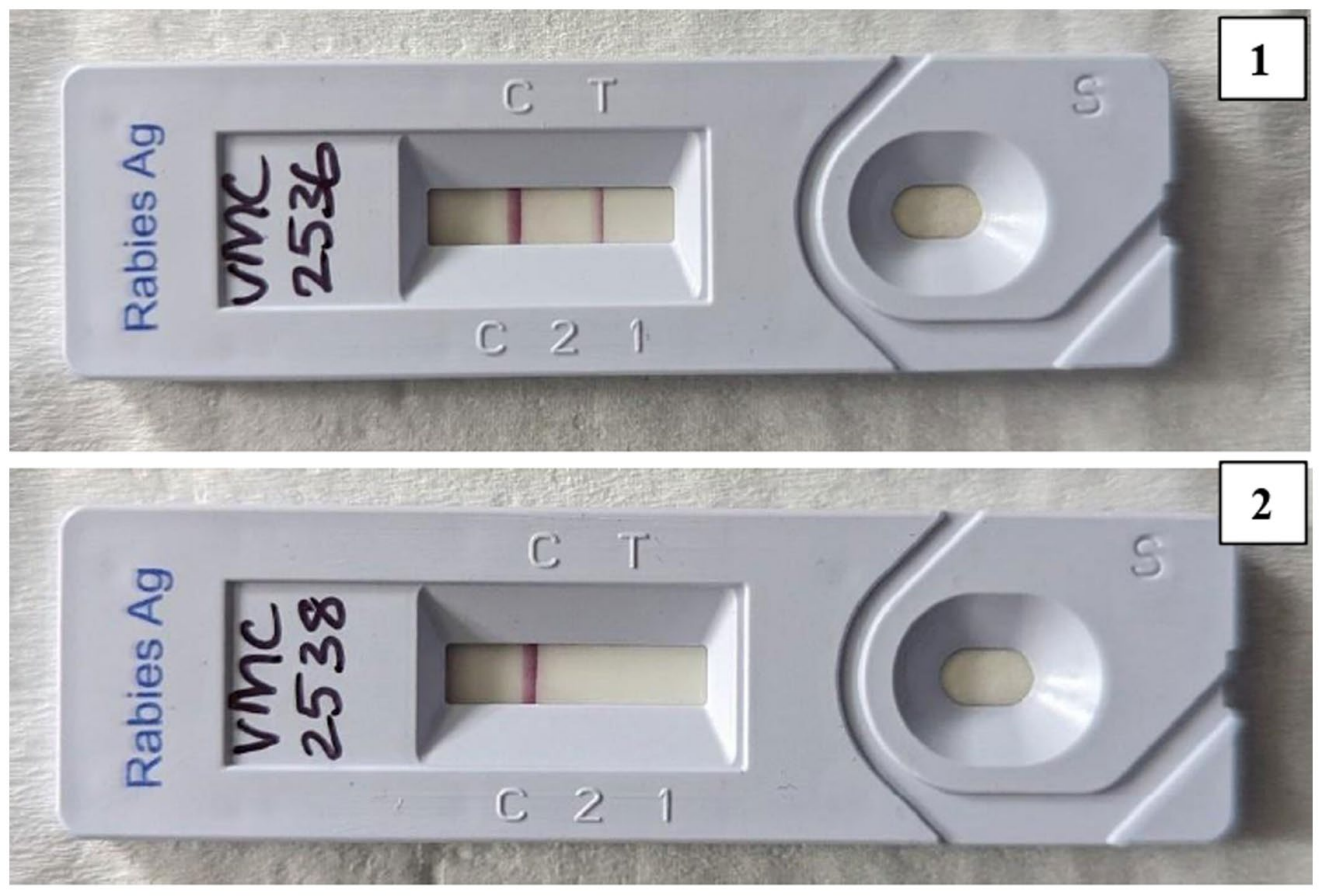
Flow immunochromatographic assay (LFA) showing a positive result (top) and a negative result (bottom).

Out of the 25 brain samples tested, 19 (76%) were found to be positive. A positive outcome was indicated by the presence of a red line at both the ‘C’ and ‘T’ positions on the device. Conversely, a red line solely at the ‘C’ position signified a negative result. The positive and negative results are shown in Figure 3.

#### 3.3.2 Direct fluorescent antibody test (DFA)

**Figure 4:**
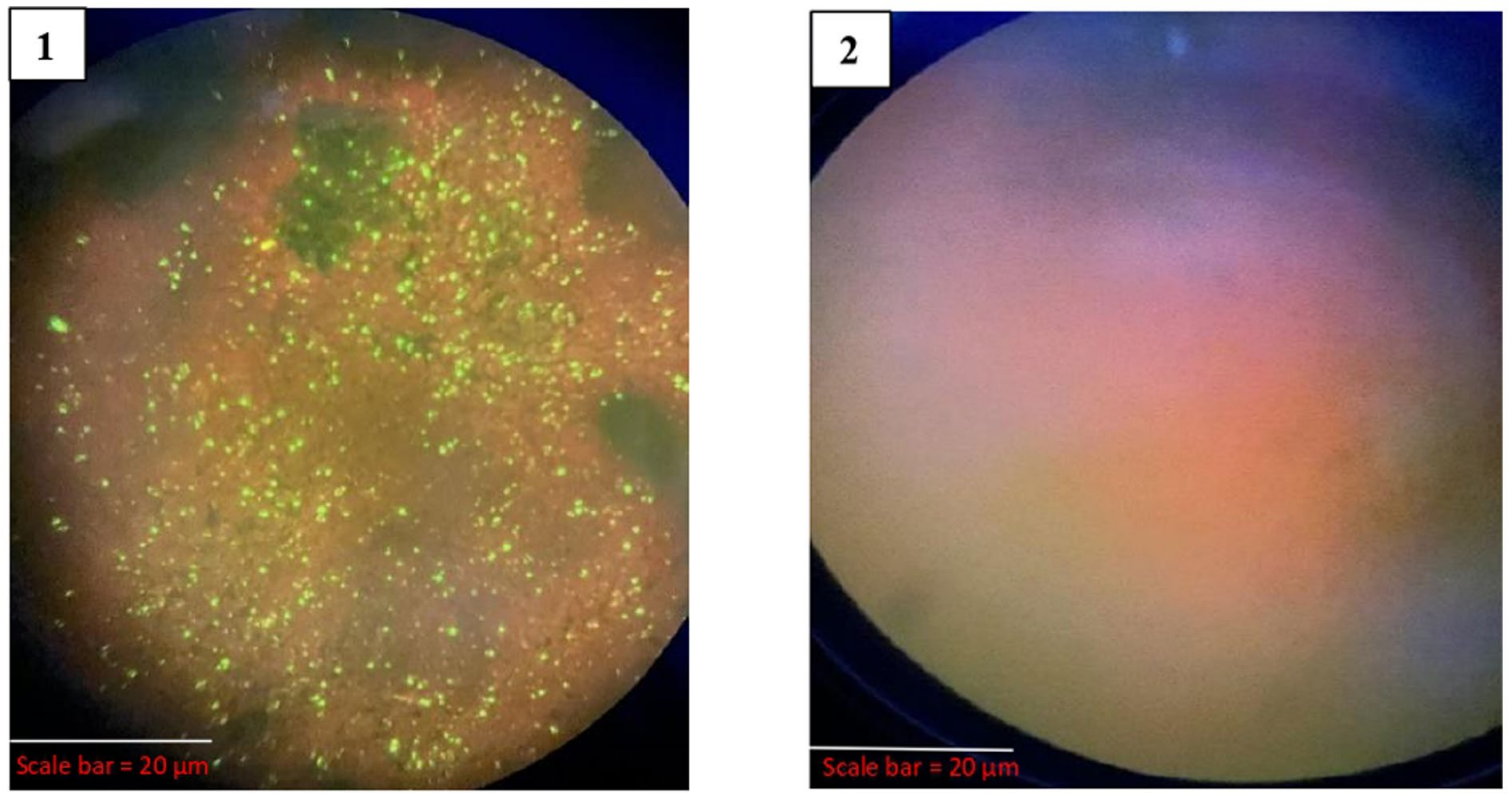
Results of DFA. 1-Positive for DFA (VMC 2683), 2-Negative for DFA (VMC 2686).

All 25 samples were assessed for the presence of viral inclusions using DFA, where 19 brain impressions (76%) exhibited a distinct apple green fluorescence, indicating the presence of rabies viral inclusions (Figure 4).

#### 3.3.3 Direct rapid immunohistochemistry test (dRIT)

**Figure 5:**
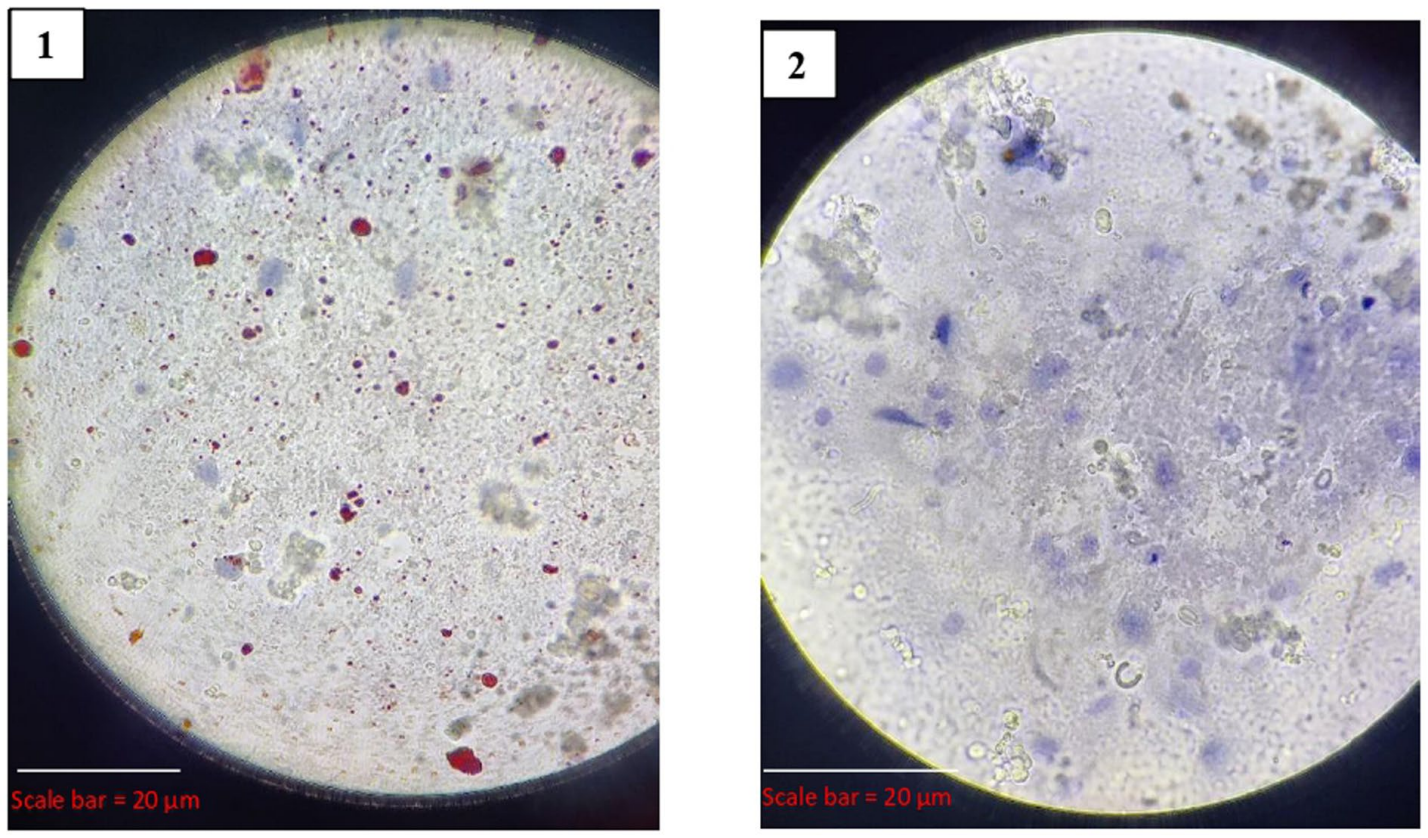
Results of dRIT. (1) Positive for dRIT (VMC 2683), (2) Negative for dRIT (VMC 2686).

Out of the 25 samples, 19 (76%) tested positive for rabies by dRIT. The impressions showing red inclusions against a blue neuronal background were considered positive. The positive and negative results are shown in Figure 5.

#### 3.3.4 One step reverse transcriptase PCR

**Figure 6:**
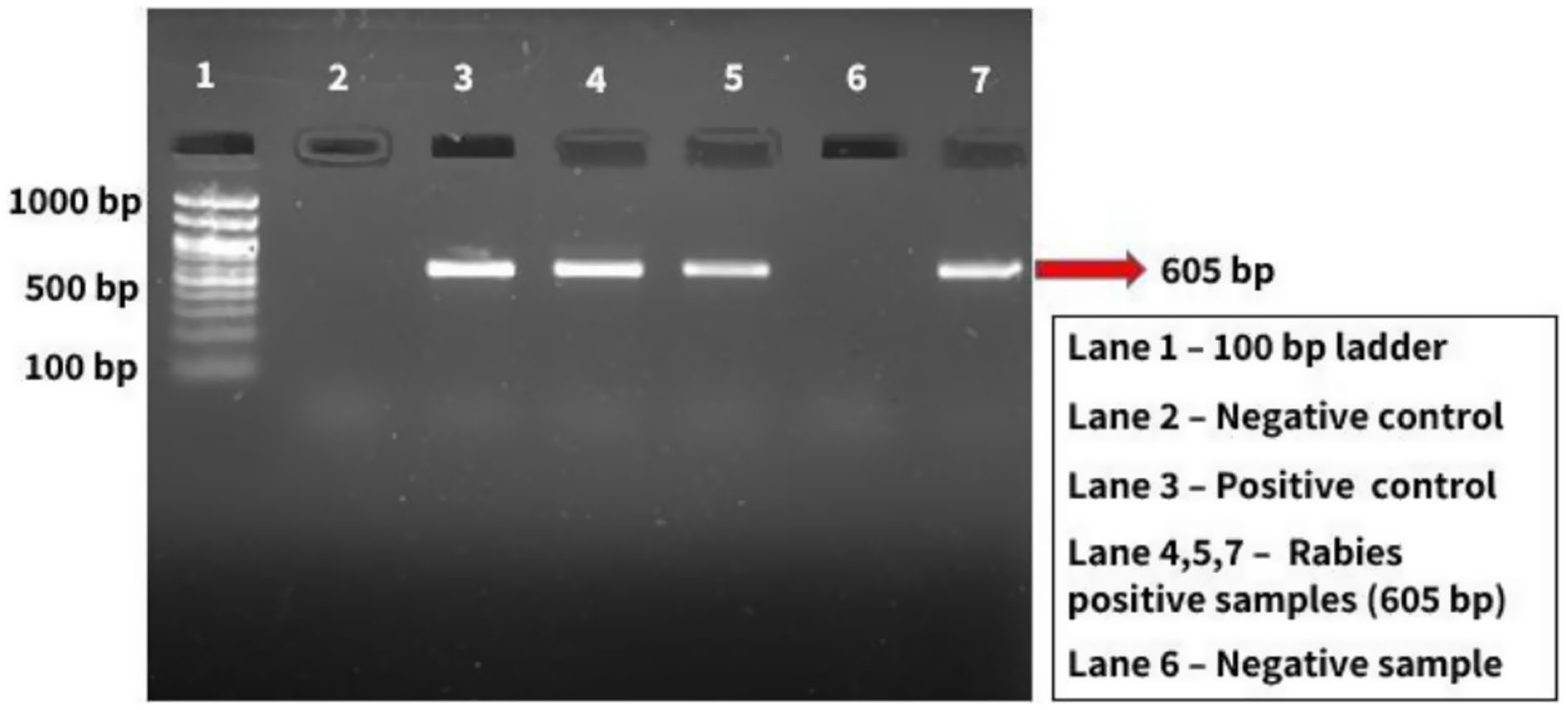
Molecular confirmation of RABV.

All the brain samples were subjected to one-step RT-PCR for the confirmation of rabies by targeting the N-gene of RABV. The PCR results confirmed that 19 (76%) of the suspected brain samples were positive for rabies. Agarose gel image visualized under UV gel documentation system, showing RABV is indicated in Figure 6.

### 3.4 Comparison of different diagnostic tests

All comparison tests (LFA, dRIT, and RT-PCR) showed 100% sensitivity and specificity when compared to the standard DFA, indicating that these diagnostic methods are equally effective in detecting Rabies virus in brain samples (95% CI for sensitivity: 82.4–100%; 95% CI for specificity: 54.1–100%, *n* = 25). Each test exhibited perfect agreement with the standard test, as summarized in Table 2.

**Table 2:** Sensitivity and specificity of comparison tests against DFA.

Comparison test	a	b	c	d	Sensitivity (%)	Specificity (%)	Total samples (N)
DFA vs. LFA	19	0	0	6	100	100	25
DFA vs. dRIT	19	0	0	6	100	100	25
DFA vs. RT-PCR	19	0	0	6	100	100	25

### 3.5 Overview of rabies cases in Puducherry

Data, including the details of age, sex, breed and locality based on laboratory diagnostic results of 25 rabies suspected cases, was analyzed to study the epidemiological attributes of dog-mediated rabies patterns in Puducherry during the study period (Supplementary Table 2). In this study, out of the 19 confirmed rabies cases, 10/19 (52.60%) involved female dogs, while 9/19 (47.60%) were male dogs. The age of the rabid dogs ranged from 2 months to 9 years. The dogs in the age group of <3 years were the most affected due to rabies, with 13/19 dogs (68.04%). All the affected dogs were found to be free-roaming dogs and lacked a proper vaccination record. The regions of Reddiarpalayam and Lawspet in the study area reported the most rabies cases, with 4 and 3 cases, respectively.

## 4 Discussion

Dog-mediated rabies has been eradicated in several regions, including Western Europe, Canada, the USA, Japan, Malaysia and some Latin American countries (17). Additionally, Australia lacks rabies among carnivores and numerous Pacific Islands have never reported rabies. In contrast, rabies continues to be a significant health concern in regions such as Africa and Asia. The disease’s clinical signs are not easily identifiable, especially early on, making diagnosis challenging due to the variable incubation period and overlapping symptoms with other conditions like transmissible spongiform encephalopathies, tetanus, listeriosis, poisoning, Aujeszky’s disease and other viral non-suppurative encephalitis, which all exhibit overt nervous signs. Notably, paralytic rabies can be confused with Guillain-Barré Syndrome (17), and a few cases of dumb rabid animals may die without exhibiting any characteristic clinical symptoms, emphasizing the importance of laboratory diagnosis. Rapid, reliable and accurate diagnosis is key to effective surveillance efforts and for effective use of post-exposure (PEP) vaccination, which are crucial for the ultimate control of this disease (9).

### 4.1 Dog bite cases in humans

Dog bites pose a significant public health concern, leading to diverse consequences on human well-being. The primary issue is the direct physical injuries, often resulting in permanent disfigurement that necessitates reconstructive surgery. Additionally, victims may also experience psychological trauma and post-traumatic stress. In severe cases, these dog bite attacks can be fatal (18).

In India, the burden of animal bites remains high, with an estimated 17.5 million incidents annually, 99% of which are attributed to dog bites. Data from the Integrated Disease Surveillance Programme (IDSP) reveal a sharp rise in these incidents, from 4.2 million in 2012 to 7.4 million in 2018 (7). Reflecting this national trend, Puducherry has experienced a marked increase in dog bite cases from 16,652 in 2020 to 20,063 in 2023. Another report notes a rise from 16,458 to 23,778 dog bite cases between 2014 and 2022 in the Puducherry region (19).

This upward trajectory in Puducherry may be attributed to multiple factors. The increase in the dog population, coupled with more frequent human-dog interactions, particularly in urban and peri-urban areas, likely contributes to the rise. Additionally, improved healthcare access and heightened awareness may lead to increased reporting and care-seeking behavior. Greater outdoor human activity, particularly among children, also raises the likelihood of encounters with dogs, thereby elevating bite risk.

Monthly analysis of dog bite cases from 2020 to 2023 in Puducherry indicates higher incidence during November (1677.5 cases) and December (1781.25 cases), with a noticeable dip in July (1331.25 cases) and August (1,291 cases). These seasonal trends align with findings from other Indian cities like Jaipur and Delhi, where higher cases during winter months have been linked to peak whelping activity and the protective behavior of female dogs toward their puppies (20, 21). However, contrasting findings from Delhi and Jammu suggest lower dog bite incidences in winter months due to reduced outdoor human activity, especially in the early mornings, and school holidays keeping children indoors (22, 23).

In summary, while global comparisons provide context, the focus should remain on region-specific dynamics in Puducherry. The seasonal and annual rise in dog bite cases underscores the need for targeted public health interventions, including community education, improved canine population control, and better surveillance to mitigate this growing public health concern.

### 4.2 Dog bite cases in animals

The present study reveals an increase in dog bite incidents among animals, escalating from 948 cases in 2022 to 1,131 cases in 2023 along with August 2023 reporting the highest number of dog bite cases in animals, with 125 cases across all species. Notably, canines constituted the majority, accounting for 54.10% (1123) of cases, with caprine and bovine species following at 30.70% (640) and 14.30% (297) in both 2022 and 2023. These trends are consistent with findings in Israel in 2002, where 94% of affected animals were dogs, followed by cats at 6% (24). Additionally, studies in Bangladesh in 2016 indicated prevalence rates of dog bites of 50% in dogs, 25.7% in goats and 14% in cattle (25). A study in 2018 reported that 77% of dogs, 15% of goats and 8% of cattle were affected due to dog bites (26). Furthermore, in 2020, the highest incidence of dog bites in animals was observed in dogs at 33.3%, followed by goats at 12.6% and cattle at 6.9% (27).

The prevalence of dog bites in dogs may stem from their territorial behavior, manifesting as aggression during feeding or fear when their territory is infringed upon. Additionally, territorial disputes arising from new entries or an overall surge in the dog population could contribute to the higher number of dog bite cases and also an increase in the number of dog bites. Comparing dog bites in goats and cattle, the higher incidence in goats may be attributed to their significantly larger population size compared to cattle, and the smaller body size of goats could make them more susceptible to dog bites (25).

When comparing the number of dog bite cases in humans and animals, bite cases were very low in animals. The strengthening of the information collection on the animal bite cases in the UT of Puducherry would give a better understanding of the status of animal bite cases.

### 4.3 Comparison of diagnostic tests

The LFA, DFA, dRIT and RT-PCR performed was compared with the results obtained.

#### 4.3.1 Lateral flow assay

Lateral Flow Assay is a pen-side test following the principle of immunochromatography. In the present study, of the 25 brain samples screened for rabies, 19 were positive and had a 100% correlation with DFA. This assay also demonstrated high sensitivity and specificity in detecting rabies from brain samples.

In agreement with the present study, a study in South Africa also reported 100% sensitivity and 100% specificity of immunochromatographic tests using virus isolates representative of all African genotypes (10) and various studies conducted using different species of brain samples collected from various states in India revealed that there is 100% correlation between DFA and LFA and also has 100% sensitivity and 100% specificity (28–30).

LFA has a lot of practical utility when it comes to diagnosing rabies at the field level. The LFA can also be used for the diagnosis using saliva, serum and blood (31). Their simplicity, portability and affordability make them ideal for use in the field without any extensive lab infrastructure. However, certain concerns surround the quality of LFA. The reliability of LFA may be compromised in diagnosing rabies in degraded animal brain samples or corneal smears, underscoring the significance of maintaining optimal sample conditions. While LFA produces rapid results, its sensitivity may vary, emphasizing the need for meticulous evaluation to ensure accurate diagnosis in different scenarios (32).

Overall lateral flow device combined with the foramen magnum method of brain sampling can potentially provide great opportunities to diagnose animal rabies in resource-limited settings and also obtain rapid results. The reliability of LFA may be compromised in degraded animal brain samples, and cost-effectiveness analysis was not performed in this study. While LFA produces rapid results, a gold standard technique is a must for confirmation; validation in larger sample sizes is needed to confirm these findings.

#### 4.3.2 Direct fluorescent antibody (DFA) test

It is considered the gold standard test for routine veterinary and human laboratory diagnosis of rabies (33). In this study, we assessed 25 brain samples for the presence of viral inclusions using the DFA, out of which 19 showed a distinct apple green fluorescence, suggesting the presence of rabies viral inclusions.

In India, several researchers have reported varying rates of overall positivity in their respective studies, reflecting regional and methodological differences. Chandranaik et al. (34) reported a positivity rate of 32.85%, while Mundas et al. (35) and Prabhu et al. (3) found higher rates of 67.54 and 64.98%, respectively. Yale et al. (15) observed a positivity of 55.56%, Chaudhary (36) reported 78%, Ghouse (28) noted 93%, Tilak (29) recorded 100%, and Govindaiah et al. (30) documented 80.65%. These findings demonstrate a broad range of positivity rates, from 32.85 to 100%. The 76% positivity rate observed in the present study aligns with this range, indicating consistency with previously documented trends across different regions and periods in India.

Collectively, these studies support the utility and reliability of DFA in detecting rabies virus in brain samples, but the constraints of this test include the requirement of an expensive fluorescent microscope, well-trained personnel, and to rule out the background autofluorescence that is produced. Moreover, the test could be subjective, suggesting that two independent readers may be necessary for routine diagnosis (37).

#### 4.3.3 Direct rapid immunohistochemistry test (dRIT)

It is a modified immune peroxidase test developed by the Centers for Disease Control and Prevention (CDC), Atlanta, USA, to detect the RABV antigen (38). This test uses highly concentrated and purified biotinylated anti-nucleocapsid monoclonal antibodies produced *in vitro* in a direct staining approach and allows a diagnosis to be made in less than 1 hour using a simple microscope. As of now, dRIT has been tested in some countries, like Africa and India (11), reporting that the sensitivity and specificity of dRIT are equivalent to those of DFA.

In the present study, of the 25 brain samples screened for rabies, 19 samples were found to be positive by dRIT. It showed a 100% correlation with DFA, indicating perfect agreement. Additionally, the dRIT demonstrated 100% sensitivity and 100% specificity, showcasing its accuracy in detecting rabies. These results are in agreement with previous studies that have evaluated the efficacy of dRIT in rabies diagnosis. A study in India conducted by Madhusudana et al. (11) observed complete agreement between dRIT and DFA in 400 brain samples. Prabhu et al. (3) tested 257 brain samples and confirmed 100% corroboration between DFA and dRIT. Ghouse (28) observed 100% agreement between dRIT and DFA in 46 brain samples from different species.

Collectively, these studies endorse dRIT as a reliable rabies diagnostic and as an alternative to DFA. It is a simple and cost-effective method that utilizes a light microscope, making it accessible in various settings, particularly in developing countries with limited resources. Additionally, dRIT provides rapid results, aiding in timely diagnosis (39).

#### 4.3.4 One-step RT-PCR

The Nucleoprotein (N) gene of the rabies virus is a highly conserved gene and hence is targeted for most immune and molecular diagnostic purposes. The present study aimed at partial N gene-based PCR. Initially, RNA was extracted from each sample and partial N gene PCR was taken up separately. Subsequently, agarose gel electrophoresis revealed amplified DNA at 605 bp.

In the present study, of the 25 brain samples screened for rabies, 19 were positive and had a 100% correlation with DFA. Additionally, the RT-PCR demonstrated 100% sensitivity and 100% specificity, showcasing its accuracy in detecting rabies. These findings are consistent with previous research on the diagnosis of rabies using molecular techniques. Araujo et al. (40) confirmed RT-PCR’s 100% accuracy in detecting rabies virus, matching DFA’s gold standard. Dandale et al. (41) reported RT-PCR and TaqMan real-time PCR had similar sensitivitiescompared to DFA. Dettinger et al. (42) in the USA further validated RT-PCR’s efficacy in confirming rabies in animal brain samples and early-stage infections, with a 100% correlation to DFA.

In India, Prabhu et al. (3) tested 257 samples and confirmed 100% corroboration between DFA and RT-PCR. Chaudhary (36) observed 100% agreement between RT-PCR and DFA in 140 samples.

Collectively, these studies endorse RT-PCR as a reliable rabies diagnostic tool, suggesting its potential as an alternative to DFA.

#### 4.3.5 Overview of rabies in Puducherry in 2023

In the present study, the distribution of rabies cases based on the sex of the animals revealed that rabies is almost equally shared between both sexes, with females at 52.6% (10/19) compared to males at 47.4% (9/19). Contrary to the present findings made by Widdowson et al. (43) in Santa Cruz, Bolivia, Gunaseelan et al. (44), Yale et al. (45) and Bharathy and Gunaseelan (46) in Chennai found that the males were the major affected sex due to rabies in all the studies. In the present study, rabies positives were encountered by non-descript dogs (100%). The percentage of non-descriptive dogs was less in the studies conducted by Gunaseelan et al. (44) (71.2%) and 76.4% by Yale et al. (45) in Chennai. Non-descript stray dogs contributed greatly to the prevalence of rabies in other dogs and humans (46).

### 4.4 Limitations of the study

This study has certain limitations that warrant consideration. The relatively small sample size, with only 19 rabies-positive cases detected in 2023, limits the statistical power of the findings and may not fully represent the true epidemiological burden of rabies in the canine population of Puducherry. Moreover, the study relied on passive surveillance, capturing only those cases that were reported by NGOs, municipal bodies, veterinary hospitals, and clinics. This approach may have led to under-reporting, as many rabid animals might have died unreported or gone undiagnosed. Additionally, the sampling was restricted to dogs suspected of rabies, introducing a degree of selection bias. Furthermore, the study did not incorporate detailed epidemiological linkages such as bite history, vaccination status, or contact tracing, which could have provided a deeper understanding of transmission dynamics and risk factors. Despite these limitations, the data represent the entirety of rabies-suspected dog cases received in the Union Territory of Puducherry during the study period and thus provide valuable baseline insights for future research and public health planning.

## 5 Conclusion

The present study highlights a significant and concerning increase in dog bite cases affecting both humans and animals in the Union Territory of Puducherry. This rising trend mirrors patterns observed in other rabies-endemic regions, suggesting a pressing public health challenge. Notably, the surge in cases during specific months points to the role of seasonal factors and heightened human-animal interactions in influencing bite incidence.

Of the 25 brain samples collected from suspected rabid dogs, 76% tested positive for rabies. Diagnostic evaluation using the Lateral Flow Assay (LFA), direct Rapid Immunohistochemical Test (dRIT), and Reverse Transcription PCR (RT-PCR) demonstrated 100% concordance with the gold-standard Direct Fluorescent Antibody (DFA) test. These findings affirm the sensitivity, specificity, and reliability of these alternative diagnostic tools. Due to their rapid turnaround times and operational simplicity, LFA and dRIT emerge as particularly valuable for field-level rabies diagnosis in resource-limited or remote settings.

Epidemiological profiling indicated that the most affected population comprised young, free-roaming, non-descript dogs, with an approximately equal distribution between males and females. These results underscore the urgent need for strengthened rabies surveillance systems, sustained mass vaccination campaigns targeting stray dog populations, and comprehensive public education initiatives focused on bite prevention and timely post-exposure prophylaxis.

In conclusion, this study provides vital insights into the epidemiology of dog bites and rabies in Puducherry. It advocates for the adoption of accessible diagnostic tools and the implementation of a One Health approach, which integrates human, animal, and environmental health strategies to mitigate rabies transmission effectively. These efforts are essential to achieving the national and global objective of eliminating dog-mediated human rabies deaths by 2030.

## Data Availability

The raw data supporting the conclusions of this article will be made available by the authors, without undue reservation.
