# Supplementary material for: First report on dog bite epidemiology and Rabies diagnosis in stray dogs: a one health study from Puducherry
Source: Front Vet Sci. 2025 Aug 29;12:1642231. doi: 10.3389/fvets.2025.1642231 (PMC12434624; doi:10.3389/fvets.2025.1642231)
Supplement: Supplementary file 1 [file Table_1.docx]

**Table 1. Veterinary Dispensaries in Puducherry included in the present study**

| **S. No** | **Veterinary Dispensary** |
| --- | --- |
| 1. | Ariyankuppam |
| 2. | Bahour |
| 3. | Madagadipet |
| 4. | Kariamanickam |
| 5. | Thirukkannur |
| 6. | Thattanchavady (Mettupalayam) |
| 7. | Villianur |
| 8. | Pondicherry |
| 9. | Sivaranthagam |

**Table 2. Details of the animal and the location of the sample**

| **S.No** | **Sample**  **No** | **Date of sample**  **collection** | **Species of**  **animal** | **Sex of the**  **animal** | **Location** |
| --- | --- | --- | --- | --- | --- |
| 1. | D1 | 04-03-2023 | Canine | Male | Reddiarpalayam |
| 2. | D2 | 11-03-2023 | Canine | Male | Kurumbapet |
| 3 | D3 | 05-05-2023 | Canine | Female | Reddiarpalayam |
| 4 | D4 | 06-05-2023 | Canine | Female | Auroville |
| 5 | D5 | 08-05-2023 | Canine | Female | Krishna Nagar |
| 6 | D6 | 16-05-2023 | Canine | Female | Auroville |
| 7 | D7 | 23-05-2023 | Canine | Male | Thavalakuppam |
| 8 | D8 | 31-05-2023 | Canine | Male | Reddiarpalayam |
| 9 | D9 | 28-06-2023 | Canine | Male | Gorimedu |
| 10 | D10 | 28-06-2023 | Canine | Male | Pondy Beach Road |
| 11 | D11 | 29-06-2023 | Canine | Male | Kathirakamam |
| 12 | D12 | 05-08-2023 | Canine | Male | Lawspet |
| 13 | D13 | 22-08-2023 | Canine | Female | Pondy Beach Road |
| 14 | D14 | 11-09-2023 | Canine | Female | Reddiarpalayam |
| 15 | D15 | 13-09-2023 | Canine | Male | Pathukannu |
| 16 | D16 | 22-09-2023 | Canine | Male | Auroville |
| 17 | D17 | 02-10-2023 | Canine | Male | Kathirakamam |
| 18 | D18 | 06-10-2023 | Canine | Female | Kurusakuppam |
| 19 | D19 | 06-10-2023 | Canine | Female | Saram |
| 20 | D20 | 06-10-2023 | Canine | Female | Pakkam X road |
| 21 | D21 | 27-10-2023 | Canine | Female | Lawspet |
| 22 | D22 | 10-11-2023 | Canine | Female | Lawspet |
| 23 | D23 | 11-11-2023 | Canine | Male | Lawspet |
| 24 | D24 | 30-11-2023 | Canine | Male | Mettupalyam |
| 25 | D25 | 01-12-2023 | Canine | Female | Lawspet |
